# Thoracic epidural anesthesia decreases endotoxin-induced endothelial injury

**DOI:** 10.1186/1471-2253-14-23

**Published:** 2014-04-05

**Authors:** Fabian Enigk, Antje Wagner, Rudi Samapati, Heike Rittner, Alexander Brack, Shaaban A Mousa, Michael Schäfer, Helmut Habazettl, Jörn Schäper

**Affiliations:** 1 Department of Anesthesiology and Operative Intensive Care Medicine, Charité–Universitätsmedizin Berlin, Campus Benjamin Franklin, Hindenburgdamm 30, Berlin 12203, Germany; 2 Institute of Physiology, Charité–Universitätsmedizin Berlin, Charitéplatz 1, Berlin 10117, Germany; 3 Department of Anesthesia and Critical Care, Josef-Schneider-Straße 2, 97080 Würzburg, Germany; 4 Department of Anesthesiology and Operative Intensive Care Medicine, Charité–Universitätsmedizin Berlin, Campus Virchow-Klinikum, Augustenburger Platz 1, Berlin 13353, Germany; 5 Department of Anesthesiology, Emergency Medicine and Critical Care Medicine, Universitätsmedizin Göttingen, Robert-Koch-Straße 40, Göttingen 37099, Germany

**Keywords:** Epidural anesthesia, Lidocaine, Endotoxemia, Endothelial injury, Cytokines, Adhesion molecules, Myeloperoxidase

## Abstract

**Background:**

The sympathetic nervous system is considered to modulate the endotoxin-induced activation of immune cells. Here we investigate whether thoracic epidural anesthesia with its regional symapathetic blocking effect alters endotoxin-induced leukocyte-endothelium activation and interaction with subsequent endothelial injury.

**Methods:**

Sprague Dawley rats were anesthetized, cannulated and hemodynamically monitored. E. coli lipopolysaccharide (Serotype 0127:B8, 1.5 mg x kg^-1^ x h^-1^) or isotonic saline (controls) was infused for 300 minutes. An epidural catheter was inserted for continuous application of lidocaine or normal saline in endotoxemic animals and saline in controls. After 300 minutes we measured catecholamine and cytokine plasma concentrations, adhesion molecule expression, leukocyte adhesion, and intestinal tissue edema.

**Results:**

In endotoxemic animals with epidural saline, LPS significantly increased the interleukin-1β plasma concentration (48%), the expression of endothelial adhesion molecules E-selectin (34%) and ICAM-1 (42%), and the number of adherent leukocytes (40%) with an increase in intestinal myeloperoxidase activity (26%) and tissue edema (75%) when compared to healthy controls. In endotoxemic animals with epidural infusion of lidocaine the values were similar to those in control animals, while epinephrine plasma concentration was 32% lower compared to endotoxemic animals with epidural saline.

**Conclusions:**

Thoracic epidural anesthesia attenuated the endotoxin-induced increase of IL-1β concentration, adhesion molecule expression and leukocyte-adhesion with subsequent endothelial injury. A potential mechanism is the reduction in the plasma concentration of epinephrine.

## Background

Due to its high prevalence and mortality in critically ill patients, sepsis is one of the major problems in clinical medicine [1,2]. During the multifactorial process of systemic inflammation, one of the prominent pathways is the activation of immune cells and endothelial cells by lipopolysaccharide, a structural component on the outer membrane of gram-negative bacteria [3]. Lipopolysaccharide, after attaching to the lipopolysaccharide binding protein, is bound to the membrane cluster of differentiation 14, and to the toll-like receptor 4 on monocytic cells and endothelial cells [4]. An intracellular signal transduction cascade leads to the synthesis of inflammatory cytokines such as tumor necrosis factor-α (TNF-α), and interleukin-1β (IL-1β), which are involved in the priming and recruitment of granulocytes, and the expression of adhesion molecules on immune cells and endothelial cells [4]. As a consequence, activated granulocytes adhere to the vascular endothelium and transmigrate into the tissue within 45 minutes [5], where they release reactive oxygen species.

Sympathetic mediators such as epinephrine and norepinephrine may induce cytokine release from immune cells [6] and can enhance migration of immune cells to sites of inflammation as well as the degree of plasma extravasation [7]. Sympathetic blockade of the adrenal medulla by thoracic epidural anesthesia (TEA) decreases the plasma concentration of epinephrine [8,9], and may, by this, modulate inflammation. In various experimental setups of systemic inflammation TEA has shown beneficial effects on microvascular perfusion [10,11], regional blood flow [8], and intestinal function [12-14]. In addition, in patients with peritonitis TEA was associated with improved gut function [15,16] and decreased length of hospital stay [16].

In this study in rats, we investigated whether TEA can modulate the endotoxin-induced endothelial injury. Effects on adhesion molecule expression were determined by immunohistochemistry and fluorescence activated cell sorting analysis. Leukocyte-endothelium interaction and extravasation were quantified by intravital microscopy and intestinal myeloperoxidase activity, respectively. Endothelial injury was quantified by wet to dry weight ratios, a surrogate marker for tissue edema.

## Methods

### Animals

After approval by the local animal care committee (Landesamt für Gesundheit und Soziales, Berlin, Germany), male Sprague–Dawley rats weighing 301 [267/319] g (median, [quartiles]) were used in all experiments. Animals were handled according to the National Institutes of Health guidelines on the use of experimental animals. The rats were housed in our animal facility under standardized conditions and had free access to standard rat chow (Altromin Spezialfutter GmbH und Co.KG, Lage, Germany) and water until the experiment.

### Surgical preparation

Epidural catheterization at the segmental level L4 was performed as described previously [17]. Epidural placement and segmental height of the catheter tip was determined after each experiment by autopsy. In preliminary experiments we measured the serum concentration of epidurally applied lidocaine 2% after epidural bolus (30 μl) and continuous infusion (30 μl/h) (n = 3 animals). serum lidocaine concentrations at one, two, three, four, and five hours of lidocaine 2%. This resulted in median serum concentrations of 0.22, 0.28, 0.21, 0.21, and 0.24 μg/ml, respectively. Following tracheostomy (PE 205, Portex) the right carotid artery and the jugular veins were cannulated (PE 50, Portex) for continuous hemodynamic monitoring.

### Experimental protocol

**Figure 1:**
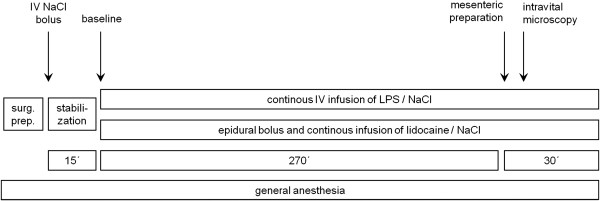
**Schematic drawing of the experimental protocol.** NaCl = sodium chloride, surg. prep. = surgical preparation, LPS = lipopolysaccharide.

The experimental protocol is shown in Figure 1. General anesthesia was with subcutaneous urethane (Urethan 99% (N), Sigma-Aldrich-Chemie GmbH, Deisenhofen, Germany; 1.5 g × kg^-1^) and intramuscular ketamine (Ketavet, Pharmacia & Upjohn GmbH, Erlangen, Germany; 50 mg × kg^-1^) in rats spontaneously breathing room air. Body temperature was kept constant at 37°C with a heating pad and a temperature probe connected to a control unit (Harvard Electronics, Edenbridge, UK). After epidural catheterization and vascular instrumentation, all animals were given an intravenous bolus injection of normal saline 0.9% (5 ml, Boehringer Ingelheim KG Delta-Pharma, Pfullingen, Germany) followed by a continuous intravenous infusion (6.5 ml × h^-1^) to maintain normovolemia throughout the experiment. Animals were then randomly allocated to one of three experimental groups: (1) a control group receiving normal saline epidurally (LPS - TEA -, n = 8), (2) an endotoxemic group receiving normal saline epidurally (LPS + TEA -, n = 8) and (3) an endotoxemic group receiving lidocaine epidurally (LPS + TEA +, n = 8). An epidural injection of 30 μl lidocaine 1% (Lidocain HCl 1%, B. Braun Melsungen AG, Melsungen, Germany) or normal saline was administered followed by a continuous epidural infusion at a rate of 30 μl × h^-1^ (Genie Plus, Kent Scientific Corporation, Torrington, CT). Simultaneously endotoxemia was induced by a continuous intravenous infusion of E. coli lipopolysaccharide (LPS, Serotype 0127:B8, Sigma-Aldrich) at 1.5 mg × kg^-1^ × h^-1^. Hemodynamic variables (mean arterial pressure, heart rate and central venous pressure) were documented 15 min after surgical preparation was finished and at regular intervals thereafter. Arterial blood was analyzed for white blood cell count (Coulter® Ac-T™ Analyzer, Beckman Coulter, Inc., Miami, FL), partial pressures of carbon dioxide and oxygen, hematocrit and acid–base status (RapidlabTM 348, Chiron Diagnostics GmbH, Fernwald, Germany) at baseline and at the end of the experiment. After 270 min of infusion of endotoxin or normal saline, a distal segment of the mesentery was prepared for intravital microscopy. After intravital microscopy, the animals were exsanguinated. Blood and organs were harvested, snap frozen in liquid nitrogen, and stored at -80°C until further analysis.

For quantification of leukocytic and endothelial adhesion molecules we investigated four and five animals per group, respectively.

### Plasma concentration of catecholamines

Norepinephrine and epinephrine plasma concentrations were determined by reversed phase high-performance liquid chromatography using a commercially available reagent kit (Biorad, Munich, Germany) as described in detail previously [10].

### Cytokine measurements

Plasma TNF-α, and IL1-β levels were quantified using a commercial enzyme-linked immunosorbent assay kit (BioSource International, Inc., Camarillo, CA). All samples were tested in duplicate. The plate was read on an automated microplate reader (Spectra Rainbow Thermo Reader and Magellan Software V 2.22, Tecan Deutschland GmbH, Crailsheim, Germany) at 450 nm. The concentrations of TNF-α and IL1-β were calculated from a standard curve and expressed in picogram per milliliter (pg/ml). The lower limit of detection for TNF-α was <4 pg/ml and for IL1-β <3 pg/ml.

### Immunohistochemistry

For immunohistochemical analysis of endothelial adhesion molecule expression, five animals per group were perfused transcardially with 60 ml phosphate-buffered saline (PBS, pH = 7.4, Biochrom AG, Berlin, Germany) and 300 ml cold PBS containing paraformaldehyde 4% (fixative solution). Ileum samples were excised, post-fixed at 4°C for 90 minutes in fixative solution and cryoprotected overnight at 4°C in PBS containing sucrose 10%. The tissues were then embedded in Tissue-Tek® O.C.T.™ compound (Miles Laboratories Inc., Elkhart, IN) and frozen at -80°C until further processing. Intestinal sections (7 μm thick) were prepared on a cryostat and mounted on gelatin-coated slides. Immunofluorescence staining of the sections was performed as described elsewhere [18]. The sections were exposed to the block solution for 1 h and then incubated with a mouse monoclonal antibody against rabbit E-selectin which cross-reacts with rat E-selectin (generously provided by B. Wolitzky, Hoffman-La Roche, Nutley, NJ), polyclonal rabbit anti-human P-selectin, which cross-reacts with rat P-selectin (1:500, BD Biosciences, Heidelberg, Germany), and a monoclonal mouse anti-rat ICAM-1 (1:500, clone number 1A129, Seikagaku, Tokyo, Japan). The sections were then incubated for 1 h with the following secondary antibodies (Vector Laboratories, Inc., Burlingame, CA): a goat anti-rabbit or horse anti-mouse antibody conjugated with fluoro-iso-thio-cyanate (FITC, 1:250). Thereafter, sections were washed with PBS, mounted in vectashield (Vector Laboratories), and viewed under a fluorescence microscope (Carl Zeiss Jena GmbH, Jena, Germany) with appropriate filters. To demonstrate specificity of staining, the following controls were included: Omission of either the primary antisera or the secondary antibodies. These control experiments did not show adhesion molecule staining. The expression of P-selectin, E-selectin and ICAM-1 was quantified by an observer, who was blinded to the experimental protocol, using a Zeiss microscope (objective: 20, eyepiece: ×10). The mean number of blood vessels in five sections per animal and ten squares (384 mm2 each) per section expressing each adhesion molecule was calculated and subsequently the absolute mean (n = five animals per group) was determined.

### Intravital microscopy

After 285 minutes a loop of the terminal ileum was exteriorized from the abdominal cavity. The mesentery was positioned on an acryl platform, covered with polyethylene foil, and constantly superfused with warmed (37°C) bicarbonate-buffered Ringer’s solution equilibrated with 5% carbon dioxide and 95% nitrogen. This experimental setup ensured permanent visualization of mesenteric vessels and prevention of accidental compression and vascular stasis. Using a Leitz microscope (Leitz, Wetzlar, Germany) equipped with a 150 W xenon lamp (XBO 150 W/1, Osram, München, Germany) and a 25 × water-immersion objective, six randomly chosen mesenteric venules were investigated. The microscopic image was transferred to a monitor (Sony, Cologne, Germany) by a video camera (SIM ICCD-05, SES GmbH, Neustadt a.d.W., Germany) and videotaped (DSR25 DVCAM, Sony, Cologne, Germany) for off-line analysis.

### Quantification of leukocyte-endothelium interaction

Off-line analysis of mesenteric venules was performed using the Vision3D software (Pries and Drüsedow, Berlin, Germany, 1998). Six single, unbranched venules with diameters of 21.15 [18.44, 25.62] μm (median [quartiles]) were examined over a length of 175 μm in each animal. Rolling of leukocytes was defined as cells marginated from the center stream and moving at a velocity significantly slower than red blood cells. Leukocyte rolling was expressed as the number of cells passing a designated point per minute per leukocyte fraction change (leucocytes × min^-1^ × μm^-2^ × leukocyte fraction change^-1^). Adhesion of leukocytes was defined as cells remaining stationary at the vessel wall for 60 seconds or longer. Adhesion was determined by counting the number of cells per 175 μm of venule length after five hours and expressed as leukocytes per endothelial surface area (leukocytes x min^-1^ × μm^-2^).

### Myeloperoxidase assay

We quantified tissue myeloperoxidase activity as a marker of neutrophil infiltration using a modified method described by Kübler et al. [19]. Briefly, frozen ileal tissues were thawed and homogenized in a potassium phosphate buffer (0.02 M, pH 7.4). After centrifugation at 4°C and 20,800 g for 15 min, the pellet was incubated for 2 h at 60°C. The pellet was then resuspended in a potassium phosphate buffer (0.05 M, pH 6.0) with 0.5% hexadecyltrimethyl ammonium bromide followed by 3 cycles of freeze-thawing and homogenization. Samples were centrifuged again at 4°C and 20,800 g for 15 min, and supernatants were stored at -80°C. Ileal myeloperoxidase activity was quantified spectrophotometrically at 450 nm, using 1.6 mM tetramethylbenzidine and 0.06 mM hydrogen peroxide. Results were expressed as units of myeloperoxidase per gram of wet tissue.

### Fluorescence activated cell sorting

All antibodies used for flow cytometry were mouse monoclonal and were purchased from Becton Dickinson GmbH (Heidelberg, Germany): anti-rat L-selectin-FITC (clone HRL1, subtype hamster IgG; 20 μg × ml^-1^), anti-rat CD11b- FITC (clone WT.5, mouse IgA 20 μg × ml^-1^) and anti-rat CD18 FITC (clone WT.3, mouse IgG_1_, 20 μg × ml^-1^). Heparinized blood samples (1 ml) were obtained via the arterial line. Aliquots of 100 μl of whole blood were left unstained or incubated with antibodies against adhesion molecules or isotype controls in the indicated concentrations (see above) for 15 min at room temperature. Cells were fixed and red blood cells lysed with fluorescence activated cell staining lysing solution (Becton Dickinson) according to manufacturer’s instructions. Cells were washed in PBS and finally stored in 250 μl PBS until analysis. At least 30,000 fluorescence activated cell staining events were collected in FACScan and analyzed using CellQuest software (PharMingen, Becton Dickinson). Granulocytes were identified by forward-sideward scatter characteristics and expression of adhesion molecules was evaluated in this subset. Isotype-matched control antibodies were as follows: hamster IgG-FITC, mouse IgG_1_-FITC and mouse IgA-FITC (all at 20 μg × ml^-1^).

### Wet-to-dry weight ratio

Standardized sections of small intestine were harvested, cleaned and weighed immediately. Then specimen were dried for 24 h at 53°C in a vacuum exsiccator and weighed again. The ratio of the weights values before and after drying was used as a measure of tissue edema.

### Statistical analysis

All tests were performed with SigmaPlot 11.0 software (Systat Software GmbH, Erkrath, Germany). A sample size calculation using the standard deviation values from previous leukocyte adhesion experiments [17] gave a required sample size of eight animals. The animals were randomized using the closed envelope protocol. Normality of data was tested by the Kolmogorov-Smirnov analysis. Parametric data are displayed as mean ± standard deviation and non-parametric data as median, quartiles and ranges. Hemodynamic data are given as mean and SEM for clear presentation. We used a one way analysis of variance (ANOVA) to determine whether there were any differences between the means of the 3 groups. For normally distributed data ANOVA was followed by an all pairwise multiple comparison procedure (Student-Newman-Keuls method). Otherwise the Kruskal-Wallis ANOVA on ranks was used, followed by an all pairwise multiple comparison procedure (Dunn’s test). Hemodynamic data were analyzed using area under the curve comparison and ANOVA on ranks. Intragroup comparisons of laboratory data were performed using the Wilcoxon signed-rank test. For all tests, statistical significance was assumed for a p < 0.05.

## Results

Segmental height of the catheter tip was determined at T 8 [2] (median [range]).

### Hemodynamic variables

**Figure 2:**
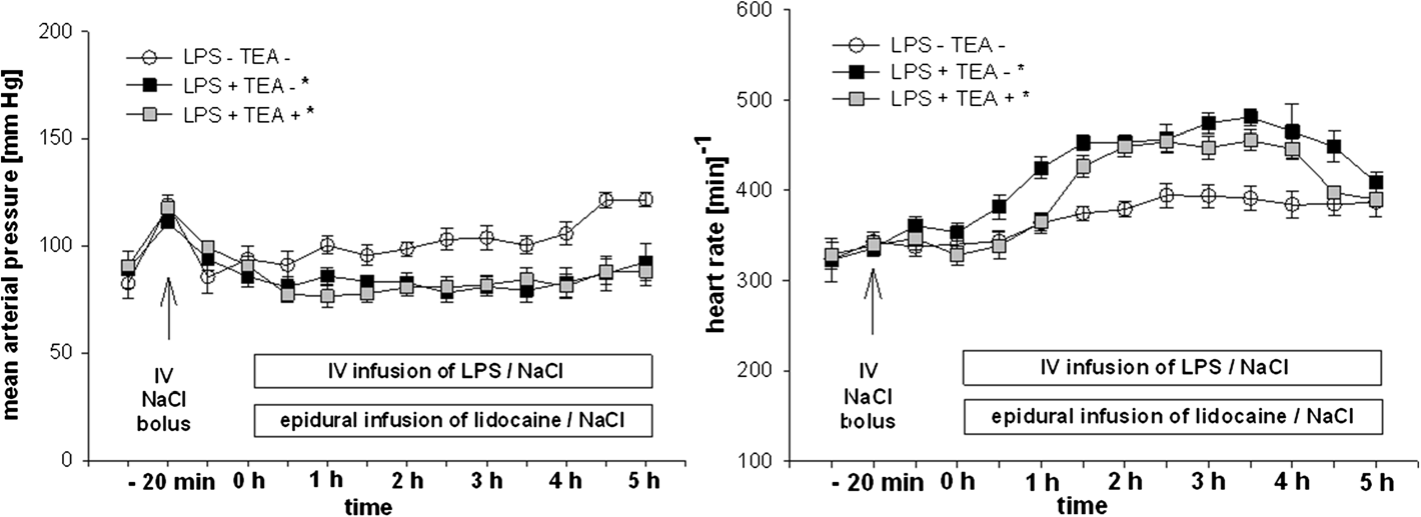
**Hemodynamic variables.** Mean arterial pressure and heart rate in endotoxemic rats with (LPS + TEA +) and without (LPS + TEA -) thoracic epidural anesthesia compared to healthy control animals (LPS – TEA -). IV = intravenous, LPS = lipopolysaccharide, NaCl = sodium chloride 0.9% solution, TEA = thoracic epidural anesthesia. Data are presented as mean and standard error of mean. * p < 0.05 versus LPS - TEA - (area under the curve, ANOVA on ranks (Dunn’s)).

Baseline values of hemodynamic variables were comparable in all groups (Figure 2). MAP values were lower in endotoxemic animals compared to healthy controls, with mean values over 80 mmHg throughout the experiment (Figure 2). Heart rate increased after infusion of lipopolysaccharide in the endotoxemic groups (Figure 2). At the end of the experimental protocol, however, the heart rate was similar in all groups (Figure 2). Central venous pressure remained unchanged in all groups throughout the experiment (data not shown).

### Laboratory variables

After five hours of lipopolysaccharide infusion both endotoxemic groups showed metabolic acidosis (Table 1). The arterial partial pressure of carbon dioxide was significantly decreased in endotoxemic rats but not in healthy controls (Table 1). Arterial oxygen partial pressure was increased in endotoxemic animals (Table 1). Base excess was significantly reduced in all groups after five hours (Table 1). Hematocrit decreased significantly in all groups (Table 1). The number of circulating leukocytes in peripheral blood was decreased at the end of the experiment compared to baseline in endotoxemic animals, but increased in controls (Table 1). The number of platelets decreased in all groups over the course of the experiment with notably lower values in endotoxemic animals compared to healthy controls (Table 1).

**Table 1:** Laboratory variables

**Variable**	**Group**	**Baseline**	**5 h LPS**	**p-value**
**pH**	LPS - TEA -	7.36 [7.33,7.38]	7.30 [7.26,7.32]	
	LPS + TEA -	7.36 [7.34,7.39]	7.30 [7.28,7.34] €	€ 0.019
	LPS + TEA +	7.37 [7.34,7.40]	7.32 [7.29,7.45]	
**p**_**a**_**CO**_**2 **_**(mmHg)**	LPS - TEA -	49 [45,51]	46 [42,50]	
	LPS + TEA -	45 [37,48]	30 [27,36] € *	€ < 0.001
	LPS + TEA +	47 [45,50]	37 [30,40] € *	€ < 0.001
**p**_**a**_**O**_**2 **_**(mm Hg)**	LPS - TEA -	72 [64,81]	89 [67,94] €	€ 0.016
	LPS + TEA -	79 [73 , 86]	101 [96 , 105] € *	€ < 0.001
	LPS + TEA +	78 [66,83]	95.5 [85,104] €	€ < 0.001
**Hematocrit (%)**	LPS - TEA -	48 [44,50]	46 [41,47] €	€ 0.024
	LPS + TEA -	47 [46,49]	36 [34,39] € *	€ < 0.001
	LPS + TEA +	49 [46,51]	36 [35,37] € *	€ < 0.001
**Base excess (mM)**	LPS - TEA -	-0.4 [-0.9,0.5]	-4.5 [-6.1,-1.9] € §	€ < 0.001
	LPS + TEA -	-1.6 [-2.7,-0.5]	-9.7 [-11.8,-7.2] €	€ < 0.001
	LPS + TEA +	0.4 [-0.5,1.7] $	-6.8 [-8.7,-4.2] € §	€ < 0.001
**Leukocytes (10**^**3**^ **μl**^**-1**^**)**	LPS - TEA -	9.5 [8.4,11.8]	18.5 [15.6,19.7] €	€ < 0.001
	LPS + TEA -	12.0 [10.6,13.9]	4.4 [3.4,5.9] € *	€ < 0.001
	LPS + TEA +	9.2 [8.0,11.7]	5.3 [3.3,6.6] € *	€ < 0.001
**Platelets (10**^**3**^ **μl**^**-1**^**)**	LPS - TEA -	995 [843,1109]	867 [724,997] €	€ 0.002
	LPS + TEA -	919 [827,1053]	120 [97,204] € *	€ < 0.001
	LPS + TEA +	941 [807,1030]	165 [94,379] € *	€ < 0.001
**Sodium (mM)**	LPS - TEA -	141 [139,143]	141 [139,143]	
	LPS + TEA -	142 [141,143]	142 [141,144]	
	LPS + TEA +	141 [140,142]	141 [140,143]	
**Potassium (mM)**	LPS - TEA -	4.4 [4.3,4.7]	4.0 [4.0,4.3] €	€ 0.003
	LPS + TEA -	4.5 [4.3,4.8]	4.6 [4.2,4.9] *	
	LPS + TEA +	4.4 [4.3,5.1]	4.3 [4.1,4.5]	

LPS = lipopolysaccharide, p_a_CO_2_ = arterial partial pressure of carbon dioxide, p_a_O_2_ = arterial partial pressure of oxygen, TEA = thoracic epidural anesthesia.

Data are median [quartiles], € p-values as depicted in the table vs. baseline, *p < 0.05 vs. LPS - TEA -; $ p < 0.05 vs. LPS + TEA -, § p < 0.05 versus LPS + TEA –.

### Plasma concentrations of catecholamines and cytokines

Plasma concentration of norepinephrine at the end of the experiment was similar in all three groups (Table 2). Plasma concentration of epinephrine at the end of the experiment was significantly higher in endotoxemic animals without TEA as compared to both other groups (Table 2).

**Table 2:** Plasma concentrations of catecholamines and cytokines

**Variable**	**LPS - TEA -**	**LPS + TEA -**	**LPS + TEA +**
Norepinephrine [ng/l]	2979 [2528,3826]	4543 [3750,5458]	5052 [2902,7252]
Epinephrine [ng/l]	293 [162,496]	901 [568,1628] #	614 [488,1078]
IL-1β [pg/ml]	29.3 [19.7,37.8]	59.9 [54.0,67.7] *	47.7 [32.0,57.5
TNF-α [pg/ml]	16.0 [14.3,17.7]	17.6 [15.7,19.4]	19.6 [18.0,25.8]

Plasma concentrations of catecholamines and cytokines at the end of the experiment. LPS = lipopolysaccharide, TEA = thoracic epidural anesthesia. IL-1β = interleukin-1β, TNF-α = tumor necrosis factor-α. Data are median [quartiles], # p < 0.05 vs. LPS - TEA – and LPS + TEA +; * p < 0.05 vs. LPS - TEA -.

When compared to healthy controls plasma concentration of IL-1β was significantly higher in endotoxemic animals without TEA but not with TEA (Table 2). Plasma concentration of TNF-α was low and comparable in all groups (Table 2).

### Endothelial adhesion molecules

**Figure 3:**
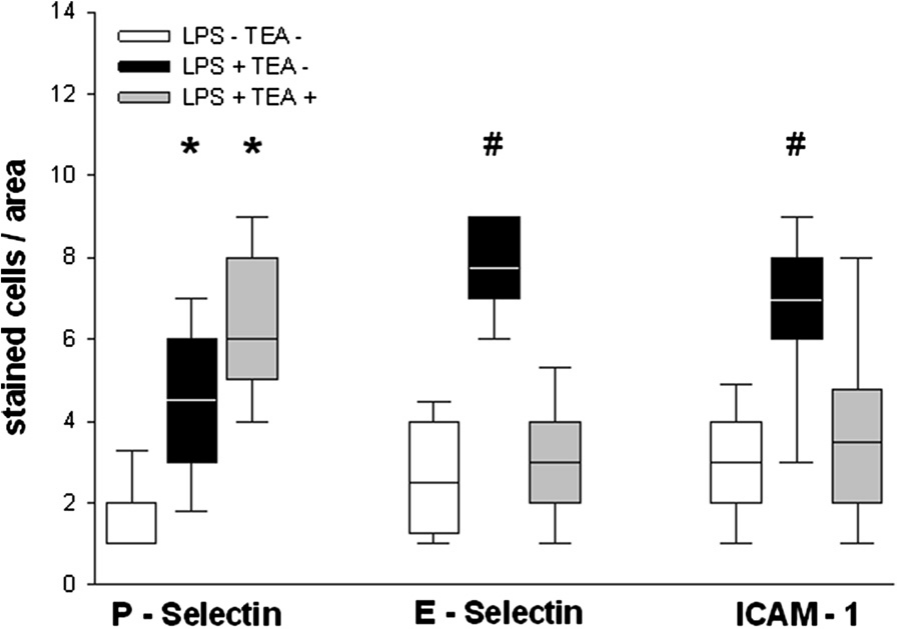
**Endothelial expression of adhesion molecules.** LPS = lipopolysaccharide, TEA = thoracic epidural anesthesia, ICAM-1 = intracellular adhesion molecule-1. Box plots represent median, quartiles and 10th/90th percentile; * p < 0.05 vs. LPS - TEA -; # p < 0.05 vs. LPS - TEA – and LPS + TEA + .

Administration of lipopolysaccharide was associated with an increase in the expression of endothelial adhesion molecules P-selectin, E-selectin, and ICAM-1. TEA significantly attenuated this effect on E-selectin and ICAM-1 (Figure 3).

### Leukocytic adhesion molecule expression

**Figure 4:**
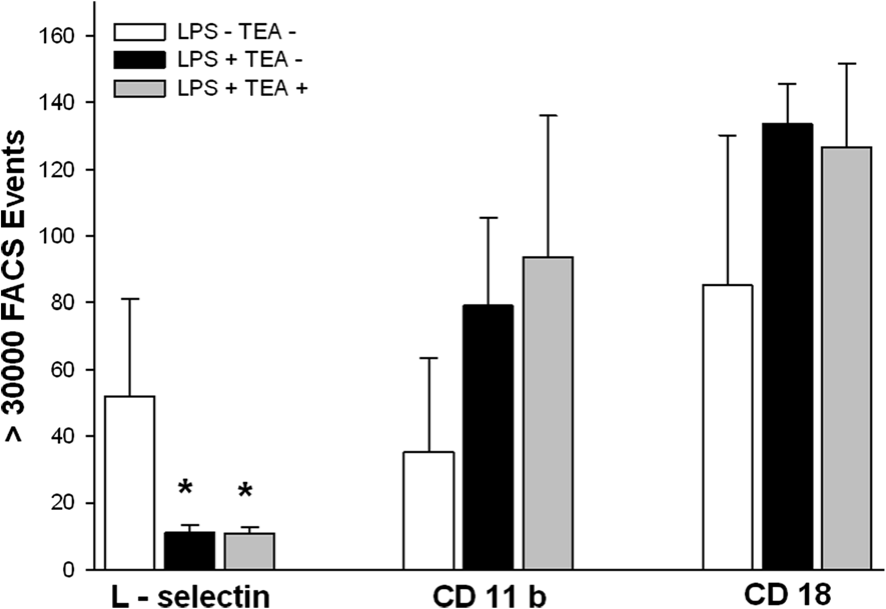
**Leukocytic adhesion molecule expression.** LPS = lipopolysaccharide, TEA = thoracic epidural anesthesia, CD = cluster of differentiation, FACS = fluorescence activated cell sorting; Data are mean and standard deviation, * p < 0.05 vs. LPS - TEA -.

The expression of L-selectin on leukocytes was lower in both endotoxemic groups when compared to healthy control animals (Figure 4). The expression of CD11b/CD18 after five hours was similar in all groups (Figure 4).

### Leukocyte-endothelium interaction

**Figure 5:**
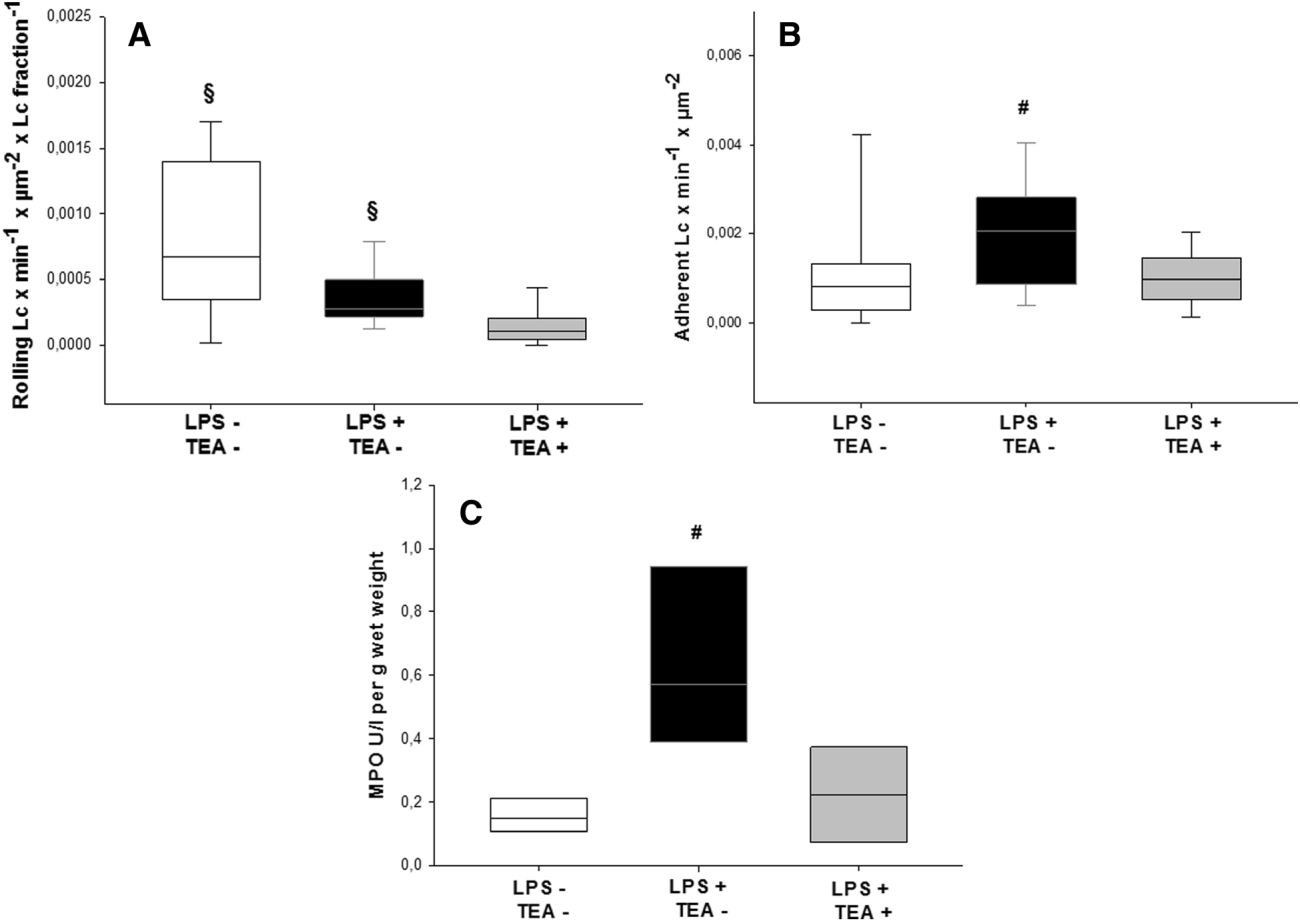
**Leukocyte-endothelium interaction.** LPS = lipopolysaccharide, TEA = thoracic epidural anesthesia, Lc = leukocytes, MPO = myeloperoxidase; Panel **A** shows rolling leukocytes, panel **B** shows adherent leukocytes, panel **C** shows the myeloperoxidase concentration in the small intestine per g wet weight. Data are median (quartiles and 10th/90th percentile), § p < 0.05 vs. LPS + TEA +; # p < 0.05 vs. LPS - TEA - and LPS + TEA + .

The extent of leukocyte rolling in mesenteric venules was highest in control animals. It was significantly lower in endotoxemic animals with TEA than in both other groups (Figure 5, panel A). The number of adherent leukocytes was highest in endotoxemic animals without TEA and also significantly reduced by TEA (Figure 5, panel B). Tissue myeloperoxidase activity in the small intestine was significantly increased in the endotoxemic group, with significantly lower values in animals with TEA (Figure 5, panel C).

### Intestinal tissue edema

**Figure 6:**
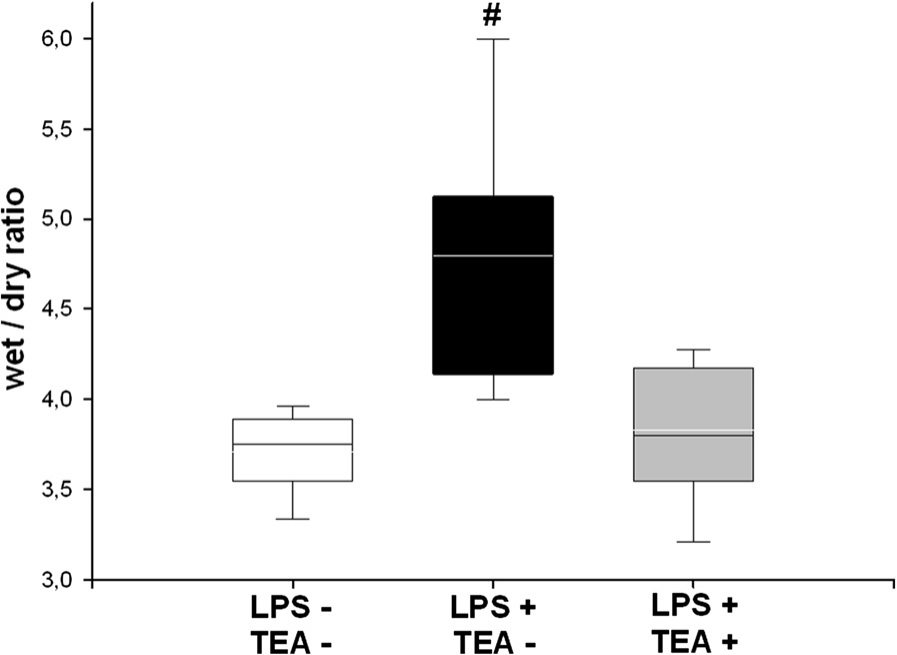
**Intestinal tissue edema.** LPS = lipopolysaccharide, TEA = thoracic epidural anesthesia; Data are median (quartiles and 10th/90th percentile), # p < 0.05 vs. LPS - TEA - and LPS + TEA +.

Wet-to-dry weight ratios of the small intestine were significantly increased in endotoxemic animals without TEA (Figure 6). The values in endotoxemic animals with TEA were similar to those in controls (Figure 6).

## Discussion

This experimental animal study investigated the effects of thoracic epidural anesthesia with regional sympathetic blockade on the lipopolysaccharide-induced endothelial injury in rats. Endotoxemic animals with TEA had lower plasma concentrations of epinephrine and IL-1β than those without TEA. This was associated with a lower expression of the endothelial adhesion molecules E-selectin and ICAM-1, less leukocyte adhesion to mesenteric venules, and decreased neutrophil accumulation in intestinal tissue. Finally, TEA was associated with less endothelial injury as quantified by intestinal tissue edema.

### Hemodynamic variables

In both endotoxemic groups, MAP decreased slightly, but did not fall below 80 mmHg after the start of the lipopolysaccharide infusion, which is consistent with a model of normotensive endotoxemia (Figure 2). Heart rate increased during the course of endotoxemia (Figure 2). This is in line with former observations [8,10,20,21], and has been correlated with an increased cardiac output in a similar rodent model [22]. Heart rate in both endotoxemic groups decreased with exteriorization of the bowel for intravital microscopy. Simultaneously, MAP slightly increased in all groups. Both phenomena are likely caused by the decrease in abdominal, and intrathoracic pressure. The fact that heart rate was similar in both endotoxemic groups, implies that the cranial spread of lidocaine is below the segments innervating the heart (T1-4). Central venous pressure values did not differ between experimental groups (data not shown), implying a similar volume status in all groups.

### Systemic variables

The endotoxin-induced decrease in base excess (Table 1) is consistent with a well-described effect on mitochondria leading to lactatic acidosis [23]. This was compensated by increased ventilation, and resulted in normal pH values after five hours of endotoxemia (Table 1). The decrease in hematocrit in endotoxemic animals (Table 1) is likely to be due to the lipopolysaccharide-induced trapping of blood cells in microvessels [23], which has been described for erythrocytes [24], leukocyctes [25], and platelets [26].

### Cytokine response

Lipopolysaccharide is known to stimulate immune cells and endothelial cells, which then release inflammatory mediators such as TNF-α and IL-1β, causing an increase in capillary permeability [27]. Moreover, catecholamines have also been shown to induce a release of inflammatory cytokines such as TNF-α and IL-1β from mouse macrophages of a similar magnitude [6]. TNF-α plasma concentration has its maximum at 60–90 min after administration of endotoxin [28] with a half-life of 30 min. This explains why at five hours after the start of endotoxin infusion, the plasma concentration of TNF-α was not significantly greater than that in control animals.

Lipopolysaccharide infusion induced a significant increase in IL-1β plasma concentrations, whereas in animals with TEA the concentrations were similar to those in controls. This is in agreement with the results of Bedirli et al., who demonstrated a reduction of IL-1β plasma concentrations and tissue damage in rats with mesenteric ischemia/reperfusion with TEA [29]. The authors, however, did not determine the plasma concentration of catecholamines in these experiments. IL-1β is known to increase the endothelial adhesion molecules ICAM-1 and E-selectin [30,31]. This represents a plausible explanation for the subsequent TEA effects on adhesion molecule expression and leukocyte-endothelium interaction.

### Adhesion molecules

The lipopolysaccharide infusion in our experiments was associated with an increase in the expression of the endothelial adhesion molecules P-selectin, E-selectin, and ICAM-1 (Figure 3). TEA significantly attenuated the expression of E-selectin and ICAM-1, but not of P-Selectin (Figure 3). Since our detection method does not differentiate between intracellularly stored and externalized P-selectin, we hypothesize that P-selectin, which is stored in endothelial Weibel-Palade bodies, was less externalized and shed less in animals with TEA. This hypothesis, however, cannot be confirmed by our results, and needs further investigation. Decreased ICAM-1 expression has also been demonstrated in rats with TEA subjected to mesenteric ischemia/reperfusion [29].

The interactions between the autonomic nervous system and the immune system are complex. While activation of the parasympathetic nervous system has a clearly anti-inflammatory effect [32], catecholamines as neurotransmitters of the sympathetic nervous system exhibit both inflammatory and anti-inflammatory dose-dependent effects on the various immune cells [33].

The catecholamine epinephrine has been shown to increase the expression of endothelial adhesion molecules in mouse hearts [34]. This may explain that accumulation of neutrophils in the lungs of healthy mice increased after epinephrine treatment [35]. Sympathetic blockade by administration of an alpha-1- and beta-adrenoreceptor antagonist attenuated the expression of ICAM-1, and leukocyte-endothelium interaction in a rat model of type 2 diabetes [36]. The same authors demonstrated that epinephrine promoted nuclear translocation of nuclear factor-κB p65, and increased expression of E-selectin and ICAM-1 in cultured endothelial cells [36]. Furthermore, acute lung injury in rats was aggravated when phagocyte-derived catecholamine production was increased, but was attenuated when production was inhibited [6]. In healthy individuals, infusion of adrenaline was associated with an increase in neutrophil respiratory burst [37]. TEA effects on endothelial adhesion molecules, therefore, may be due to the reduction of epinephrine concentration by sympathetic blockade of the adrenal gland, and may represent an important mechanism in the modulation of inflammation.

L-selectin expression on leukocytes significantly decreased with endotoxin infusion (Figure 4). This is in line with the well-described phenomenon of L-selectin shedding after neutrophil activation with chemotactic factors [38]. Leukocyte expression of CD11b and CD18 was not different among groups in our experiments (Figure 4). This may be due to the late blood sampling after five hours of endotoxemia, because the lipopolysaccharide-induced expression of CD11b and CD18 integrins on circulating leukocytes has been shown to reach a maximum as early as 30 minutes after the inflammatory stimulus [39].

### Leukocyte-endothelium interaction and myeloperoxidase tissue activity

The number of rolling leukocytes in mesenteric venules was higher in controls (LPS - TEA -), and endotoxemic animals without TEA (LPS + TEA -) than in endotoxemic animals with TEA (LPS + TEA +, Figure 5A). Surgical stress alone, therefore, appears to be sufficient to induce leukocyte rolling, which was not further increased by endotoxin. TEA, however, had an anti-inflammatory effect and reduced the number of rolling leukocytes significantly (Figure 5A). For adherence and extravasation of leukocytes a more intense stimulus was needed: in endotoxemic animals without TEA the number of adherent (Figure 5B) and extravasated (Figure 5C) leukocytes increased significantly, whereas adherence and extravasation in animals with TEA was comparable to control animals, again implying an anti-inflammatory effect of TEA on endothelium and leukocytes.

We postulate that the TEA effects on endothelial adhesion molecules directly affect leukocyte-endothelium interaction, and tissue accumulation of granulocytes. In models of mesenteric ischemia/reperfusion, ICAM-1 blockade almost completely abolished the increase in leukocyte–endothelium interaction in intestinal vessels [40] and myeloperoxidase activity [41]. Recruitment of circulating leukocytes and migration of leukocytes into the inflamed tissue is a crucial step in the pathogenesis of inflammatory organ damage. In response to endotoxin [42] or cytokines [43], neutrophils release large quantities of reactive oxygen species through respiratory burst, which is essential for killing invading microorganisms but also increases tissue damage [44]. Our results, therefore, suggest that TEA attenuates the cellular inflammatory response to endotoxin and may also have beneficial effects on subsequent tissue damage.

### Endothelial permeability and tissue edema

Endotoxin-induced endothelial permeability with subsequent tissue edema was significantly decreased by TEA (Figure 6). This is in line with other investigations, which found less intestinal tissue edema, and fewer histological signs of tissue injury in animals with TEA subjected to endotoxemia [13] or ischemia/reperfusion [29].

### Limitations of the study

The results of animal experiments or in vitro investigations do not necessarily match with the results of clinical investigations. Especially the interaction between catecholamines and cytokine release of immune cells underlies various influencing factors. In humans, epinephrine infusion prior to LPS injection suppressed TNF-alpha release and increased the plasma concentration of anti-inflammatory IL-10 [45]. In patients undergoing esophagectomy, extensive continuous sympathetic blockade (C3-L2) prevented the intraoperative increase in epinephrine plasma concentration, but did not influence the increase in IL-1 plasma levels. Similarly, in patients with coronary bypass surgery, TEA did not reduce the plasma concentration of soluble circulating ICAM-1 [46]. Therefore, the results of our experiments cannot be simply translated into the clinical setting. On the other hand, our study provides new implications to explain the positive effects of TEA on the recovery of patients with peritoneal sepsis [16].

We chose the intravenous administration of E. coli endotoxin as a model of inflammation because standardization and reproducibility is higher than that achieved by coecal ligation and puncture models. Furthermore, we intended to avoid manipulation of the gut as an influencing factor on the variables. To achieve an increase in epithelial permeability we modified the model by administering LPS continuously [21] instead of increasing a bolus dose.

A major concern was that systemic effects of epidurally applied lidocaine could be responsible for the TEA effects on mediator release, expression of adhesion molecules and leukocyte-endothelium interaction. Systemic application of lidocaine is known to reduce endothelial injury in response to endotoxemia [47,48]. The mechanisms underlying the protective effects of lidocaine remain unclear. Current literature postulates a dose-dependent inhibition of granulocytes with respect to substance release, and migration to the site of action [49]. There is evidence that systemic application of lidocaine suppresses the synthesis of proinflammatory cytokines/chemokines such as IL-1β [50]. Continuous intravenous administration of lidocaine attenuated the increase of leukocyte adherence in mesenteric venules of endotoxemic rats [25] and inhibited neutrophil recruitment to the site of inflammation in rabbits with peritonitis [51]. Studies evaluating the effects of lidocaine on leukocytic adhesion molecules indicate an inhibitory effect on the expression of L-selectin and CD11b/CD18 integrins [50,52,53]. All authors, however, reported higher dosages and measured several-fold higher plasma concentration of lidocaine compared to the concentration of 0.33 μg/ml in former experiments with a similar setup and epidural infusion of lidocaine 2% [8]. We cannot with any certainty exclude the possibility that the systemic distribution of lidocaine is another supporting mechanism for the effects of TEA, nevertheless, the dosage of lidocaine in our study remained below the effective level described in literature.

## Conclusion

In a rat model of endotoxin-induced endothelial injury, regional sympathetic blockade by means of TEA was associated with lower concentrations of inflammatory cytokines, less endothelial activation and leukocyte adhesion, and a decrease in intestinal neutrophil accumulation and tissue edema. The reduction in plasma epinephrine concentration by TEA represents a plausible explanation, while systemic effects of the local anesthetic as a further potential mechanism could not be excluded.

### Key messages

Thoracic epidural anesthesia decreased endotoxin-induced endothelial permeability in intestinal vessels. This could be due to a decrease in epinephrine plasma concentrations with a consequent reduction in leukocytic and endothelial activation and interaction.

## Abbreviations

TNF-α: Tumor necrosis factor-α; IL-1β: Interleukin-1β; TEA: Thoracic epidural anesthesia; LPS: Lipopolysaccharide; PBS: Phosphate-buffered saline; FITC: Fluoro-isothiocyanate; MAP: Mean arterial pressure; ICAM-1: Intercellular adhesion molecule-1; CD: Cluster of differentiation; FACS: Fluorescence activated cell sorting.

## Competing interests

The authors declare that they have no competing interests.

## Authors’ contributions

FE carried out the animal experiments, performed intravital microscopy, cytokine measurements, quantification of myeloperoxidase, statistical analysis, and drafted the manuscript. AW helped with animal experiments and cytokine measurements. RS helped with cytokine measurements and quantification of myeloperoxidase. HR and AB performed FACS analysis. SM carried out immunohistochemistry. MS and HH participated in the design of the study, the discussion of results, and helped to draft the manuscript. JS designed the study, was involved in animal experiments, intravital microscopy, cytokine measurements, statistical analysis and manuscript preparation. All authors read and approved the final manuscript.

## Pre-publication history

The pre-publication history for this paper can be accessed here:

http://www.biomedcentral.com/1471-2253/14/23/prepub
